# Electrocardiography vs. Auscultation to Assess Heart Rate During Cardiac Arrest With Pulseless Electrical Activity in Newborn Infants

**DOI:** 10.3389/fped.2018.00366

**Published:** 2018-11-27

**Authors:** Deandra H. Luong, Po-Yin Cheung, Megan O'Reilly, Tze-Fun Lee, Georg M. Schmolzer

**Affiliations:** ^1^Faculty of Science, University of Alberta Edmonton, AB, Canada; ^2^Centre for the Studies of Asphyxia and Resuscitation, Royal Alexandra Hospital Edmonton, AB, Canada; ^3^Department of Pediatrics, Faculty of Medicine and Dentistry, University of Alberta Edmonton, AB, Canada

**Keywords:** infants, newborn, neonatal resuscitation, asphyxia, heart rate, electrocardiography, auscultation

## Abstract

**Background:** In 2015, the neonatal resuscitation guidelines incorporated the use of electrocardiography (ECG) to monitor heart rate of newborns. However, previous studies have indicated that cardiac arrest with pulseless electrical activity rhythm (PEA) may occur in the delivery room, rendering this method problematic.

**Objective:** To evaluate the accuracy of ECG and auscultation to assess heart rate during PEA.

**Methods:** A total of 45 piglets (age 1–3 days, weight 1.7–2.3 kg) were exposed to 30 min normocapnic alveolar hypoxia followed by asphyxia until asystole, achieved by disconnecting the ventilator and clamping the endotracheal tube. During asphyxia, heart rate (HR) was assess using auscultation, ECG, and carotid blood flow (CBF). At the time of asystole (defined as zero CBF) HR auscultated using a neonatal/infant stethoscope was compared to ECG traces.

**Results:** The median (IQR) duration of asphyxia was 325 (200–491) s. In 8 (18%) piglets, CBF, ECG, and auscultation identified asystole. In 22 (49%) piglets no CBF and no audible heart sounds, were observed, while ECG displayed a HR ranging from 17 to 75/min. Fifteen (33%) piglets remained bradycardic (defined as HR of < 100/min) after 10 min of asphyxia, which was identified by CBF, ECG, and auscultation. The overall accuracy of ECG and auscultation in the detection of HR were 51 and 80%, respectively (*p* = 0.004).

**Conclusion:** In cases with PEA ECG is not superior in correctly identifying HR in newborn piglets.

## Introduction

Immediately after birth, a newborns heart rate (HR) is assessed to determine the effectiveness of spontaneous respiratory effort and the need for subsequent interventions (1, 2). Changes in a newborn's HR are considered the most sensitive indicator of effectiveness for each intervention. Therefore, identifying a rapid, reliable, and accurate method to measure the newborn's HR is critically important (1, 2). Until 2015, auscultation of the precordium was recommended as the preferred physical examination method, and pulse oximetry was recommended as an adjunct to provide a non-invasive, rapid, and continuous assessment of HR during resuscitation (1, 2). Studies comparing clinical assessment with pulse oximetry reported that auscultation or palpation underestimates HR by −14 and −21 beats per minute, respectively, suggesting they are both unreliable and inaccurate (3). Also, several studies reported that a ECG displayed a reliable HR faster than pulse oximetry, and pulse oximetry tended to underestimate the newborn's HR and might have led to potentially unnecessary interventions (4–7). However, most of the included newborns did not require resuscitation, and very few required chest compressions. Therefore, ECG to assess HR in newborns who require chest compression should be approached with caution.

In this of particular importance as we recently described that cardiac arrest with pulseless electrical activity (PEA) was present in ~50% of neonatal piglets exposed to hypoxia and asphyxia (8). Cardiac arrest with PEA, defined as the presence of electrical activity without any associated mechanical activity is causes by hypoxia, hyper-/hypokalemia, hypovolemia, hypothermia, hydrogen ions (acidosis), tension pneumothorax, cardiac tamponade, thrombosis (coronary and pulmonary), and toxins (9–11). Although cardiac arrest with PEA rhythm can consist of both wide and narrow QRS-complexes, the causes of these patterns vary. PEA with narrow QRS-complexes stem from mechanical conditions whilst wide complex PEA results from metabolic problems (9–11). In adults, cardiac arrest with PEA rhythm occurs in 35–40% of in-hospital arrests, and 22–30% of out-of-hospital arrests (9–11). However, information regarding PEA in newborns is lacking. A case series of two preterm infants reported cardiac arrest with PEA due to hypocalcaemia after administration of fresh frozen plasma (12). In addition, a study in newborn piglets reported 43% of asphyxiated piglets experienced cardiac arrest with PEA, with ECG indicating a HR between 15 and 80 beats per minute (8, 13, 14).

We aimed to determine the accuracy of auscultation and ECG compared to carotid blood flow during cardiac arrest with PEA in asphyxiated piglets. We hypothesized that auscultation is superior to ECG to assess HR during cardiac arrest with PEA.

## Methods

This is a secondary analysis of two unpublished randomized controlled animal studies (These studies examined 18, 21, or 100% oxygen during either 3:1 Compression: Ventilation ratio or CC+SI (Chest compression + Sustained Inflation) during cardiopulmonary resuscitation (both studies are currently under peer review). The original studies were conducted in accordance with Animal Research Reporting of *In vivo* Experiments (ARRIVE) guidelines (13), and approved by the Animal Care and Use Committee (Health Sciences) University of Alberta (AUP00001764, AUP00002151). For this secondary analysis, we included 45 newborn mixed breed piglets (1–3 days of age, weighing 1.7–2.3 kg) from the original studies.

Piglets were instrumented following the induction of anesthesia using isoflurane, piglets were then intubated via a tracheotomy, and pressure-controlled ventilation (Acutronic Fabian HFO; Hirzel, Switzerland) was initiated at a respiratory rate of 16–20 breaths/min and pressure of 20/5 cm H_2_O. Oxygen saturation was kept within 90–100%, glucose levels and hydration were maintained with an intravenous infusion of 5% dextrose at 10 mL/kg/hr. The piglet's body temperature was maintained in the normal range of 38.5–39.5°C using an overhead warmer and water heated pads. During the experiment anesthesia was maintained with intravenous propofol 5–10 mg/kg/h and morphine 0.1 mg/kg/hr. Additional doses of propofol (1–2 mg/kg) and morphine (0.05–0.1 mg/kg) were administered as needed (14).

### Study protocol, data collection, and analysis

All piglets had the right common carotid artery exposed and enclosed with a real-time ultrasonic flow probe (2 mm; Transonic Systems Inc., Ithaca, NY), and HR was continuously measured and recorded using ECG (Hewlett Packard 78833B monitor, Hewlett Packard Co., Palo Alto, CA). This setup allowed us to simultaneously monitor HR via ECG and carotid blood flow during the experiments (14). Hypoxia was induced by exposing piglets to 30 min of normocapnic alveolar hypoxia at a fractional inspired oxygen concentration of 0.10. Hypoxia was then followed by asphyxia until asystole, achieved by disconnecting the ventilator and clamping the endotracheal tube. The study protocol further specified that either after 10 min of asphyxia or after asystole chest compression will be initiated. Cardiac arrest was defined as zero carotid blood flow (CBF). TO assess cardiac arrest one investigator used a neonatal/infant stethoscope (3M™ Littmann® Classic II Infant Stethoscope, U.S.; GMS (*n* = 28) or PYC (*n* = 17). The investigator was blinded to the ECG or the CBF flow display. Once the investigator confirmed cardiac arrest (= unable to hear a heartbeat) a marker was placed within the LabChart program (ADInstruments, Dunedin, New Zealand) to indicate time of cardiac arrest. The marker was then compared to waveforms from the ECG and CBF to determine HR at the time of cardiac arrest by auscultation. The data are presented as mean ± SD for normally distributed continuous variables and median (IQR) when the distribution is skewed. The data were tested for normality and analyzed using Stata (Intercooled 10, Statacorp, Texas, USA). The rates of accuracy and predictive values in the detection of HR by ECG and auscultation were compared by *z*-test.

## Results

We studied 45 piglets; the median (IQR) duration of asphyxia was 325 (200–491) s. In total, the piglets were asystolic in 30 cases. Eight asystolic piglets (18%) were lacking heart sounds, CBF, and an ECG HR. In 22 (49%) cases, CBF and auscultation indicated asystole but were accompanied by an ECG HR of 17–75 beats per minute. In the other words, 22 of 30 (73%) piglets had PEA as demonstrated by the presence of ECG HR but zero CBF and inaudible HR. Fifteen (33%) piglets remained bradycardic and had a HR > 100 beats per minute after 10 min of asphyxia, as indicated by auscultation, ECG and CBF. The overall accuracy of ECG and auscultation in the correct detection of HR (asystole and bradycardia) were 51 and 80%, respectively (*z* = −2.8937; *p* = 0.004). Predictive values, sensitivity and specificity are presented in Table 1. In the detection of asystole as indicated by no carotid blood flow, the sensitivity of auscultation was significantly higher than that by ECG (100 vs. 27%; *z* = −7.1925, *p* < 0.0001).

**Table 1:** Accuracy of ECG and auscultation when compared to the gold standard carotid blood flow during cardiac arrest with pulseless electrical activity.

	**No carotid flow**	**Carotid flow seen**	**Predictive values**
**ELECTROCARDIOGRAPHY (ECG)**			
Asystole on ECG	8	0	Positive 100%
HR present on ECG	22	15	Negative 41%
	Sensitivity 27%	Specificity 100%
**AUSCULTATION**			
No heart sounds	30	9	Positive 77%
Heart sounds heard	0	6	Negative 100%
	Sensitivity 100%	Specificity 40%

## Discussion

In this translational study using a newborn piglet model equivalent to a human infant at 36–38 weeks' gestation (15, 16), we found that ECG was in agreement with CBF in only 27% of cases. In 49% of the piglets, ECG displayed a heart rate of 17–75 beats per minute whilst CBF was absent and the HR inaudible. These results are similar to those found in a previous study involving asphyxiated piglets during which auscultation was 100% accurate and ECG falsely displayed a HR of 15 to 80 bpm in 43% of piglets (8). This presence of ECG activity without a detectable pulse is called cardiac arrest with PEA (9–11). The results from this study suggest that compromised infants might have a higher risk of developing PEA over other rhythms such as bradycardia or asystole. We believe that hypoxia and hypovolemia, are the most common causes of PEA in newborn infants (8). The Adult Advanced Cardiovascular Life Support defines cardiac arrest with PEA rhythm as the occurrence of cardiac electrical activity with no associated mechanical activity. Cardiac arrest with PEA rhythm might be sinus, atrial, junctional, or ventricular in origin, and is generally categorized as narrow QRS-complex (70% of cases) or wide QRS-complex PEA (10). Narrow QRS-complex PEA occurs when a significant pathophysiologic event has impaired the ability of the cardiovascular system to perfuse the body. Typically, this may be due to cardiac tamponade, pulmonary embolism, tension pneumothorax, or mechanical lung hyperinflation. In contrast, wide QRS-complex PEA represents primary electromechanical uncoupling of the myocytes and is more likely to be due to a metabolic condition (e.g., hyperkalaemia), left ventricular failure (due to ischemia), or agonal rhythm (clinically regarded as asystole with equivalent treatment approach) (9). PEA may also be caused by hypovolemia, tachydysrhythmias, and cardiomyopathy, and only a very small percentage are caused by asphyxia (17). In adults, cardiac arrest with PEA rhythm occurs in approximately 35–40% of in-hospital arrest and 22–30% of out-of-hospital cardiac arrest (11). Furthermore, cardiac arrest with PEA rhythm is associated with a poor prognosis, with a survival to discharge rate between 2 and 5% for out-of-hospital cardiac (11).

If neonatal healthcare providers observe an ECG HR but the infant remains unresponsive, they should suspect cardiac arrest with PEA and proceed with the appropriate resuscitation steps (1, 2). The current neonatal resuscitation algorithm states that chest compressions should be initiated once HR falls/remains below 60 beats per minute. However, during cardiac arrest with PEA ECG might display a HR < 60 beats per min and therefore might delay resuscitation efforts. Therefore, ECG should be used in a combination with other assessment methods including auscultation or palpation and pulse oximetry. In addition, novel methods including Doppler ultrasound or digital stethoscope have similar set-up times an accuracy compared to an ECG (18, 19). However, these technologies have only be studied in healthy newborn infants and not in compromised infants.

Our use of a piglet asphyxia model is a great strength of this translational study, as this model closely simulates delivery room events, with the gradual onset of severe asphyxia leading to cardiac arrest (15, 16). However, several limitations should be considered: Our asphyxia model uses piglets that have already undergone the fetal to neonatal transition, and piglets were sedated/anesthetized. Furthermore, our model requires piglets to be intubated with a tightly sealed endotracheal tube to prevent any endotracheal tube leak; this may not occur in the delivery room as mask ventilation is frequently used (20, 21). All these factors may affect the occurrence of PEA in the model, in addition to species differences. Nevertheless, our findings are still clinically relevant as cardiac arrest with PEA most likely are caused by hypoxia/asphyxia. Regardless, PEA is an area of concern in the delivery room and the limitations of using ECG should be further investigated.

## Conclusion

During cardiac arrest with PEA ECG overestimates HR compared to carotid blood flow and auscultation. Therefore, ECG should be used in combination with other assessment methods including auscultation, palpation, and/or pulse oximetry.

## Author contributions

GS, P-YC, T-FL, MO: Conception and design; GS, P-YC, T-FL, MO, DL: Data collection, data analysis and interpretation, drafting of the article, critical revision of the article for important intellectual content, final approval of the manuscript.

### Conflict of interest statement

The authors declare that the research was conducted in the absence of any commercial or financial relationships that could be construed as a potential conflict of interest.
